# Genome Sequencing and Analysis of the Tasmanian Devil and Its Transmissible Cancer

**DOI:** 10.1016/j.cell.2011.11.065

**Published:** 2012-02-17

**Authors:** Elizabeth P. Murchison, Ole B. Schulz-Trieglaff, Zemin Ning, Ludmil B. Alexandrov, Markus J. Bauer, Beiyuan Fu, Matthew Hims, Zhihao Ding, Sergii Ivakhno, Caitlin Stewart, Bee Ling Ng, Wendy Wong, Bronwen Aken, Simon White, Amber Alsop, Jennifer Becq, Graham R. Bignell, R. Keira Cheetham, William Cheng, Thomas R. Connor, Anthony J. Cox, Zhi-Ping Feng, Yong Gu, Russell J. Grocock, Simon R. Harris, Irina Khrebtukova, Zoya Kingsbury, Mark Kowarsky, Alexandre Kreiss, Shujun Luo, John Marshall, David J. McBride, Lisa Murray, Anne-Maree Pearse, Keiran Raine, Isabelle Rasolonjatovo, Richard Shaw, Philip Tedder, Carolyn Tregidgo, Albert J. Vilella, David C. Wedge, Gregory M. Woods, Niall Gormley, Sean Humphray, Gary Schroth, Geoffrey Smith, Kevin Hall, Stephen M.J. Searle, Nigel P. Carter, Anthony T. Papenfuss, P. Andrew Futreal, Peter J. Campbell, Fengtang Yang, David R. Bentley, Dirk J. Evers, Michael R. Stratton

**Affiliations:** 1 Wellcome Trust Sanger Institute, Hinxton, CB10 1SA, UK; 2 Illumina Cambridge Ltd., Chesterford Research Park, Little Chesterford, Essex CB10 1XL, UK; 3 Comparative Genomics Group, Research School of Biological Sciences, Australian National University, Canberra 2601, Australia; 4 Bioinformatics Division, The Walter and Eliza Hall Institute of Medical Research, Parkville, Victoria 3052, Australia; 5 Department of Medical Biology, University of Melbourne, Parkville, Victoria 3010, Australia; 6 Illumina Hayward, 25861 Industrial Boulevard, Hayward, CA 94545, USA; 7 Menzies Research Institute Tasmania, University of Tasmania, Private Bag 23, Hobart, Tasmania 7001, Australia; 8 Animal Health Laboratory, Department of Primary Industries, Parks, Water and Environment, PO Box 46, Kings Meadows, Tasmania 7249, Australia; 9 European Bioinformatics Institute, Wellcome Trust Genome Campus, Hinxton CB10 1SA, UK; 10 Department of Mathematics and Statistics, University of Melbourne, Parkville, Victoria 3010, Australia

## Abstract

The Tasmanian devil (*Sarcophilus harrisii*), the largest marsupial carnivore, is endangered due to a transmissible facial cancer spread by direct transfer of living cancer cells through biting. Here we describe the sequencing, assembly, and annotation of the Tasmanian devil genome and whole-genome sequences for two geographically distant subclones of the cancer. Genomic analysis suggests that the cancer first arose from a female Tasmanian devil and that the clone has subsequently genetically diverged during its spread across Tasmania. The devil cancer genome contains more than 17,000 somatic base substitution mutations and bears the imprint of a distinct mutational process. Genotyping of somatic mutations in 104 geographically and temporally distributed Tasmanian devil tumors reveals the pattern of evolution and spread of this parasitic clonal lineage, with evidence of a selective sweep in one geographical area and persistence of parallel lineages in other populations.

**PaperClip:**

## Graphical abstract

**Graphical Abstract:**

**Figure:**
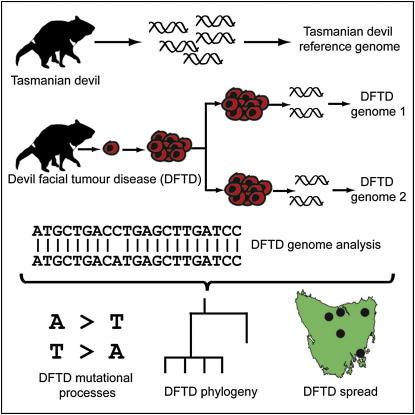

**Highlights:**

► Whole-genome sequences of the Tasmanian devil and two distant cancer subclones ► The Tasmanian devil cancer lineage originated recently in a female devil ► The devil cancer genome is relatively stable despite ongoing evolution ► Clonal divergence and geographic spread elucidated through patterns of mutation

## Teaser

Whole-genome sequences of the Tasmanian devil and two devil cancer subclones suggest that the cancer first arose from a female devil and that the clone has subsequently genetically diverged during its spread across Tasmania.

## Introduction

Cancers are clonal cell lineages that arise due to somatic changes that promote cell proliferation and survival. Although natural selection operating on cancers favors the outgrowth of malignant clones with replicative immortality, the continued survival of a cancer is generally restricted by the life span of its host. Tasmanian devil facial tumor disease (DFTD) is an unusual cancer that has survived beyond the death of the individual that spawned it by acquiring adaptations for transmission between hosts. This cancer has spread through the Tasmanian devil population and is threatening the species with extinction (Hawkins et al., 2006; McCallum et al., 2009). The genomes of the Tasmanian devil and its transmissible cancer, DFTD, are thus of interest both from the perspective of conservation of a threatened species as well as for the insights they may provide into the origins, somatic evolution and population genetics of an extraordinarily divergent neoplastic clonal lineage.

The Tasmanian devil (*Sarcophilus harrisii*) is a marsupial carnivore endemic to the island of Tasmania, Australia. Tasmanian devils are solitary nocturnal scavengers that weigh up to 12 kg and generally live for 5 or 6 years in the wild (Owen and Pemberton, 2005). They are seasonal breeders and females rear a maximum of four pouch young each year (Owen and Pemberton, 2005). The species has limited genetic diversity, although three genetically distinct geographically defined subpopulations have been described (Jones et al., 2004; Miller et al., 2011).

**Figure 1:**
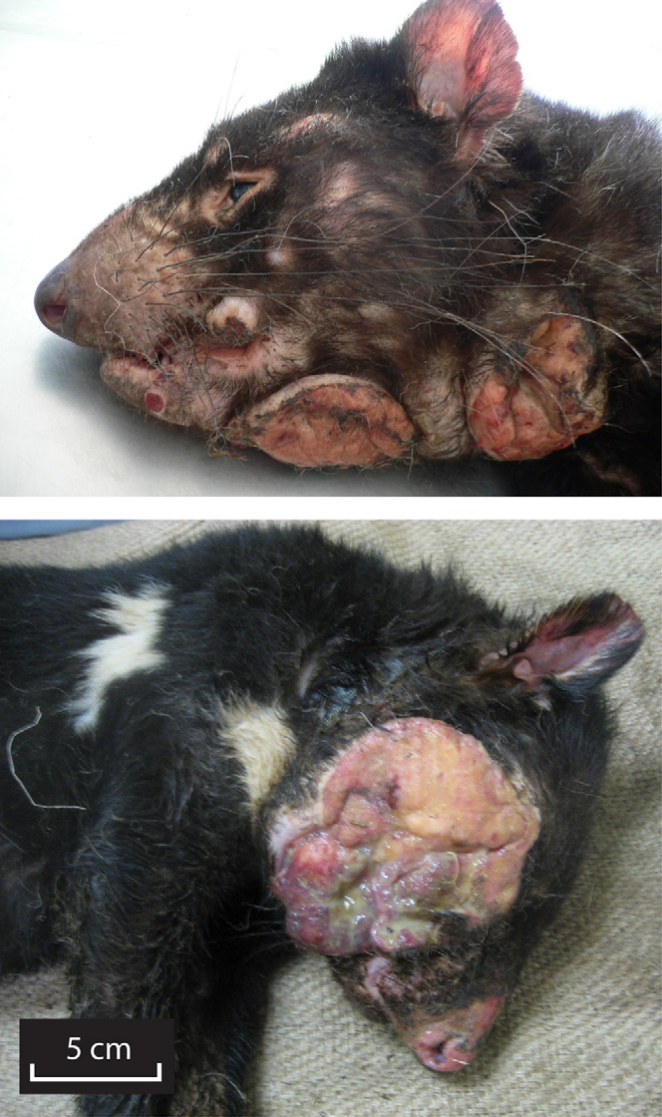
Tasmanian Devil Facial Tumor Disease Tasmanian devil facial tumor disease (DFTD) is a single cancer lineage spread by the horizontal transfer of living cancer cells.

DFTD was first observed in northeastern Tasmania in 1996 (Hawkins et al., 2006). The disease is characterized by the appearance of tumors, usually on the face and inside the mouth of affected animals, which frequently metastasise and usually cause death within months (Figure 1) (Hawkins et al., 2006; Lachish et al., 2007; Loh et al., 2006a). Most commonly observed in sexually mature individuals of 2 years or older, DFTD occurs equally in male and female devils (Hawkins et al., 2006; Loh et al., 2006a). The cancer has spread rapidly through the Tasmanian devil population and has been associated with devil population decline (Hawkins et al., 2006; Lachish et al., 2007). Epidemiological studies have documented the expansion of the disease down the east coast of Tasmania and its continuing progression toward the west coast (Hawkins et al., 2006; Lachish et al., 2007).

DFTD spreads by the direct transfer of living cancer cells, usually through bites inflicted on the face during mating and feeding interactions (Hamede et al., 2008; Pearse and Swift, 2006). DFTD is believed to be of neural crest origin, and the cancer cells express a number of genes of the Schwann cell lineage (Loh et al., 2006b; Murchison et al., 2010). The mechanism whereby the clone avoids immune rejection during colonization of allogeneic hosts remains unknown. Although low genetic diversity may contribute to DFTD susceptibility, experiments have indicated that devils are normally capable of mounting immune reactions to allogeneic grafts (Kreiss et al., 2011; Siddle et al., 2007).

DFTD is one of two known naturally occurring clonally transmissible cancers, the other being the canine transmissible venereal tumor (CTVT) of dogs (Murchison, 2009). CTVT is a sexually transmitted lineage that is found around that world and that may have first arisen thousands of years ago from the cells of a wolf or East Asian breed of dog (Murgia et al., 2006; Rebbeck et al., 2009). Genetic analysis of the global diversity of CTVT cancers has indicated that the lineage has achieved considerable heterogeneity, with substantial lineage diversity present worldwide (Murgia et al., 2006; Rebbeck et al., 2009). Divergence time estimates suggest that modern CTVT may represent a recent global sweep of an ancient lineage (Rebbeck et al., 2009). In contrast to CTVT, little is known of the population diversity of DFTD or the dynamics of its spread through its host population.

Cancer genomes are characterized by somatic changes including single-base substitution mutations, small insertions and deletions (indels), structural rearrangements, and copy number alterations. Analysis of the catalog of somatic mutations in cancer genomes can lead to greater understanding of the mutational events that triggered clonal outgrowth and the exposures or DNA repair defects that were responsible for the mutations in the first place (Pleasance et al., 2010a, 2010b). DFTD is a transmissible clone that has spread through the devil population in a process similar to metastasis. Its widely divergent lineage as a malignant clone make it an almost unique model for studying the genomic stability and long term evolution of cancer cells.

We have sequenced, assembled, and annotated the normal genome of the Tasmanian devil, and we have used this reference to analyze the genomes of a second normal Tasmanian devil and two geographically distant DFTD cancer subclones. In addition, we have analyzed the genetic diversity present in 104 DFTD tumors collected from distant locations throughout Tasmania over a period of 7 years. Our analysis has led to the identification of genetic features of the original devil that gave rise to DFTD, a description of the underlying mutational processes that have characterized DFTD progression, annotation of gene variants that may have contributed to DFTD pathogenesis, and a map of the clonal dynamics of the disease during its spread through Tasmania.

## Results

### The Tasmanian Devil Genome

To generate a reference genome for the Tasmanian devil, we sequenced and assembled the genome of a 5-year-old female Tasmanian devil. Sequencing libraries were prepared from genomic DNA extracted from a cell line derived from normal fibroblasts. These were sequenced from both ends, yielding 2.87 × 10^9^ pairs of 100 bp sequence reads. Additional “mate pair” libraries, produced by circularising genomic DNA fragments of between 3 kilobase pairs (kb) and 10 kb in length, were generated and 50 bp was sequenced from both ends in order to assist with genome assembly.

**Figure S1:**
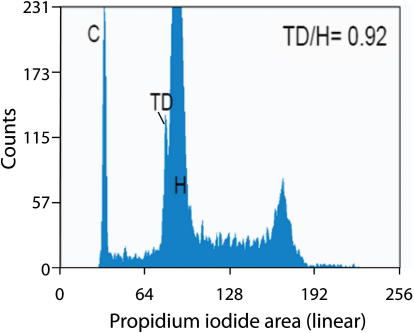
Comparative Genome Size in Tasmanian Devil, Human and Chicken, Related to Table 1 Univariate plot of a mixed nuclear suspension containing chicken erythrocyte nuclei, Tasmanian devil leukocyte nuclei and human leukocyte nuclei stained with propidium iodide. The DNA content is displayed as a histogram. DNA analysis revealed three separate peaks on the plot; C (chicken erythrocyte nuclei), TD (Tasmanian devil) and H (human). The ratio of Tasmanian devil to human DNA content is displayed inset on the plot.

The genome was assembled with the Phusion2 assembly pipeline (Mullikin and Ning, 2003), and assembly features are summarized in Table 1. We estimated the size of the Tasmanian devil genome to be between 2.89 and 3.17 gigabase pairs (Gb) using both sequencing and flow cytometry data (Table S1 and Figure S1 available online). This is comparable with previous estimates of the Tasmanian devil and other marsupial genome sizes (Mikkelsen et al., 2007; Miller et al., 2011; Renfree et al., 2011). The Tasmanian devil genome has a G+C content of 36.4%, similar to that of the opossum (37.8%) but lower than that of humans (45.2%).

**Figure S2:**
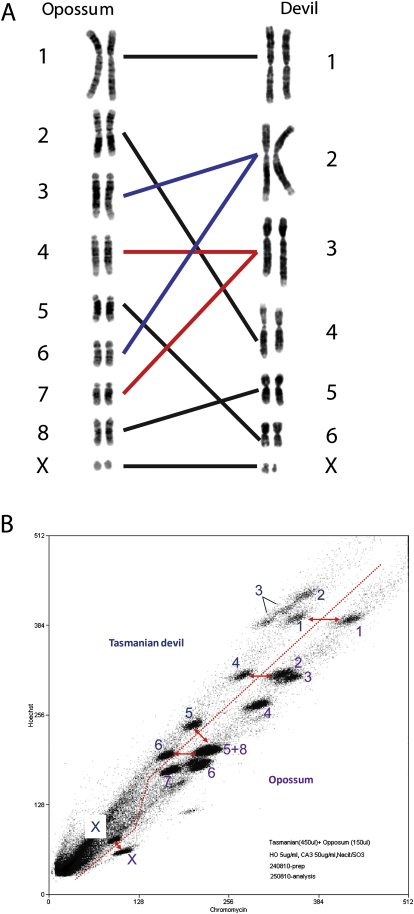
Cross-Species Cytogenetic Comparison between Devil and Opossum, Related to Figure 2 (A) Chromosome correspondence between Tasmanian devil and opossum established by hybridizing Tasmanian devil chromosome-specific paint probes onto opossum metaphases. Lines connect each devil chromosome with the opossum chromosome with which it predominantly hybridized. Devil chromosomes 2 and 3 were each homologous to two opossum chromosomes, illustrated with blue and red lines respectively. (B) Flow cytometry analysis of Tasmanian devil and opossum chromosomes. The red line marks the division between Tasmanian devil chromosomes (above the line) and opossum chromosomes (below the line). This shift toward the Hoechst axis in the Tasmanian devil chromosomes relative to opossum indicates greater A+T content in Tasmanian devil. Peaks for chromosomes with one to one correspondence between devil and opossum are connected with red arrows.

To determine the chromosomal locations of our assembled contigs, we individually sorted each of the seven Tasmanian devil chromosomes from the female devil fibroblast cell line using a flow cytometer. Fifty thousand copies of each devil chromosome were collected, amplified, and sequenced. Alignment of the chromosome reads with the assembled contigs was used to assign the contigs to chromosomes; in addition, this method was used to detect and correct assembly errors by identifying contigs with homology to more than one chromosome. Using this method, we were able to assign 35,534 supercontigs (99%) to chromosomes. The number of bases assigned to each chromosome correlated with the flow cytometry measurement of chromosome DNA content (Table S2). Tasmanian devil chromosomes were named according to the system described by Pearse and Swift (Pearse and Swift, 2006), which differs in the naming of chromosomes 1 and 2 to a previous karyotype nomenclature for devils (Eldridge and Metcalf, 2006; Martin and Hayman, 1967). We used cross-species chromosome painting to determine the homology between devil and opossum chromosomes (Figure S2). We then used conservation with the opossum genome as a template for ordering supercontigs on each devil chromosome.

Tasmanian devil genes were identified using the Ensembl genome annotation pipeline (Curwen et al., 2004; Potter et al., 2004), modified to incorporate devil transcriptome data. In total 18,775 protein-coding gene models were constructed, 1,213 of which did not have orthologs in the human or opossum genomes. We specifically searched the Tasmanian devil genome for a set of 451 genes that have been causally linked with cancer in humans (Futreal et al., 2004). Orthologs for 398 of these genes could be detected in the Tasmanian devil genome. Three hundred sixty-three microRNAs (miRNAs) were annotated in the Tasmanian devil genome by aligning small RNA sequence reads (Murchison et al., 2010). One hundred nineteen predicted miRNAs were devil orthologs of previously identified miRNAs and 244 were predicted novel miRNAs.

### Tasmanian Devil Cancer Genome Landscape

**Figure 2:**
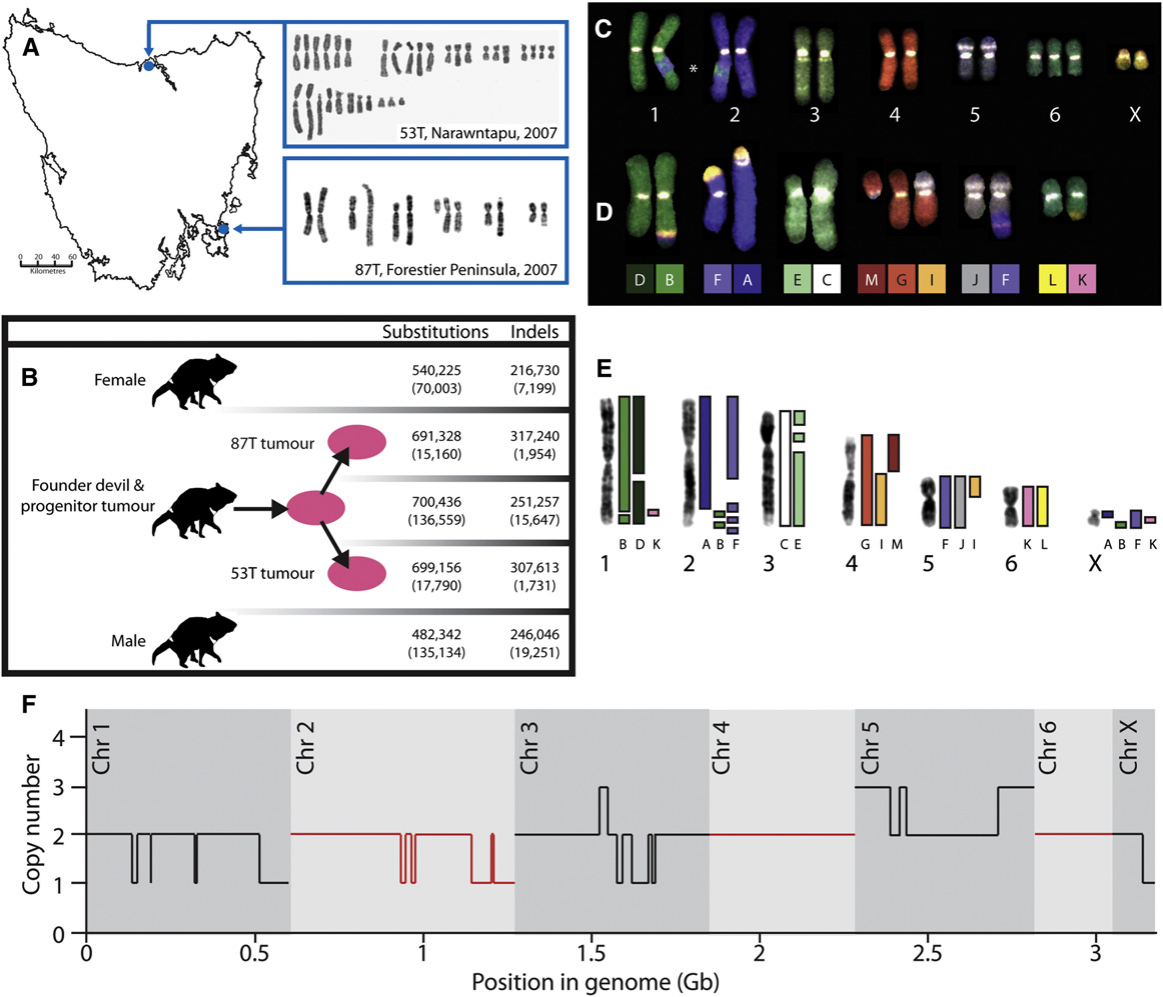
Variation in Tasmanian Devil Normal and Cancer Genomes (A) Location, year of isolation, and karyotypes for 87T and 53T DFTD cancer cell lines. (B) Four genomes were sequenced in this study, two normal Tasmanian devil genomes (female and male) and two DFTD cancer genomes (87T and 53T). DFTD originated in the DFTD founder devil, and 87T and 53T are both clonally derived from their most recent common ancestor tumor (the progenitor tumor). The female normal sequence was used to assemble the Tasmanian devil reference genome. The number of substitutions and indels compared with the reference sequence is indicated for each genome. The number of variants that were unique to each genome is indicated in brackets. The number of variants in the most recent common ancestor tumor was inferred using the variants that were common between 87T and 53T. (C) Forward chromosome painting for the normal female fibroblast cell line carrying trisomy 6 that was used to generate the reference genome assembly. ^∗^ indicates a region of overlap between chromosomes 1 and 2 that was present in the metaphase image that was used to generate the karyotype. Cytogenetic comparison between Tasmanian devil and opossum is summarized in Figure S2. (D) Forward chromosome painting for the 87T DFTD tumor. (E) Reverse painting was performed by flow sorting 87T chromosomes to produce paints (labeled A to G and I to M) and hybridizing these with normal Tasmanian devil metaphases. The F paint includes two similarly sized 87T chromosomes that we were unable to separate with flow cytometry. (F) Summary of copy number variation in 87T DFTD genome (including only changes >10 Mb in size). See Figure S3 for complete 87T and 53T copy number data. See also Figures S2 and S3.

We conducted cytogenetic analyses and sequenced the genomes of two DFTD cell lines, 87T and 53T, from geographically different regions of Tasmania (Figure 2A). 87T is derived from a tumor from a devil captured in 2007 in southeast Tasmania, and 53T was established in 2007 from a lung metastasis in a devil from the north coast of Tasmania.

**Figure S3:**
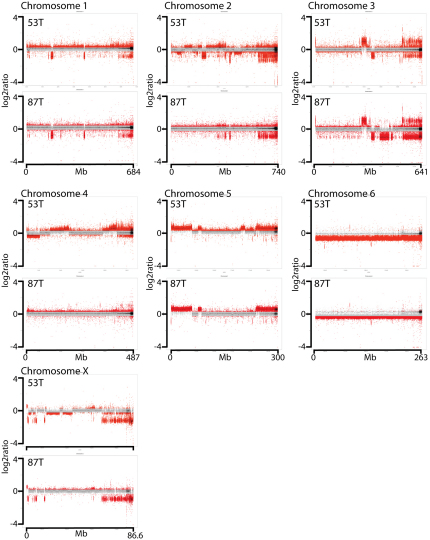
Genomic Copy Number Variation in 87T and 53T, Related to Figures 2, 3, and 4 Separate plots are shown for each chromosome (chromosomes 1 to X). Each dot represents the log2 ratio between the number of sequence reads in the tumor genome and the number of sequence reads in the female normal genome that align within a 2 kb genomic window. If p < 1 × 10-5, dot is red, otherwise dots are gray. Windows which contained no reads in the tumor (i.e., putative homozygous deletions) are represented with a red dot at log2 ratio of −4.

Alignment of the DFTD cancer cell line genomes with the reference genome yielded 691,328 and 699,156 single base substitutions in 87T and 53T, respectively, and 317,240 and 307,613 indels in 87T and 53T, respectively (Figure 2B). The number of variants in the DFTD genomes was somewhat higher than the number of variants observed in the normal female devil, and in a second normal male genome sequenced to assess normal variation (Figure 2B). This is not surprising, given that the DFTD genome contains variants that were present in the constitutional genome of the devil that first gave rise to the DFTD clone (the founder devil), as well as somatic variants that have arisen since DFTD has been a malignant clonal lineage (Figure 2B). These estimates are consistent with previous studies (Miller et al., 2011). Cytogenetic analyses indicated that the two DFTD subclones have differences in their karyotypes (Figure 2A). 87T is pseudodiploid with 13 chromosomes, whereas 53T is pseudotetraploid with 32 chromosomes. We used labeled flow-sorted chromosomes derived from a normal devil cell line as probes for forward chromosome painting of 87T. This experiment revealed several cytogenetic changes in DFTD (Figures 2C and 2D). Reverse chromosome painting, using labeled DNAs derived from flow-sorted chromosomes from 87T, provided further insights into the translocations in 87T and revealed heterozygous deletions on chromosomes 1, 2, and 3, as well as trisomy 5p (Figure 2E). Copy number analysis indicated that 87T has few detectable hemizygous deletions and no detectable high-level amplifications (Figure 2F and Figure S3).

These analyses indicate that the DFTD genome contains substitutions, indels, copy number changes, and rearrangements. We next devised methods to identify subsets of variants of germline origin (i.e., those that were present in the constitutional genome of the founder devil) and those of somatic origin (i.e., those that arose during clonal proliferation of the DFTD lineage) in order to investigate the origin and somatic evolution of the DFTD clone.

### Origin of DFTD

DFTD was first observed in 1996 in northeast Tasmania (Hawkins et al., 2006). Previous studies have indicated that the cancer is derived from the cells of one devil (the DFTD founder), and has subsequently spread through the devil population as a clone (Pearse and Swift, 2006). We do not have DNA from the founder's normal genome, as this animal was a wild Tasmanian devil that lived and probably died prior to 1996. However, variants from this devil's constitutional genome remain within the DFTD cells that make up the tumors of thousands of devils.

We sought to reconstruct the genome of the founder devil by searching for common variants between the genomes of the two DFTD subclones, 87T and 53T. These variants will include normal variation that was present in the founder's genome as well as somatic variants in DFTD that arose prior to divergence of the 87T and 53T lineages. We found 700,436 common single base substitutions and 251,257 common indels between 87T and 53T (Figure 2B). At least 563,877 single-base substitution variants and 235,610 indels are likely to be the founder's germline variants, as we also found them in either the female or male normal devil genomes. The remaining 136,559 substitutions and 14,647 indels will include private germline variants that were specific to the founder devil and not found in the two normal genomes that we sequenced as well as somatic mutations that have been acquired by the DFTD lineage.

**Figure 3:**
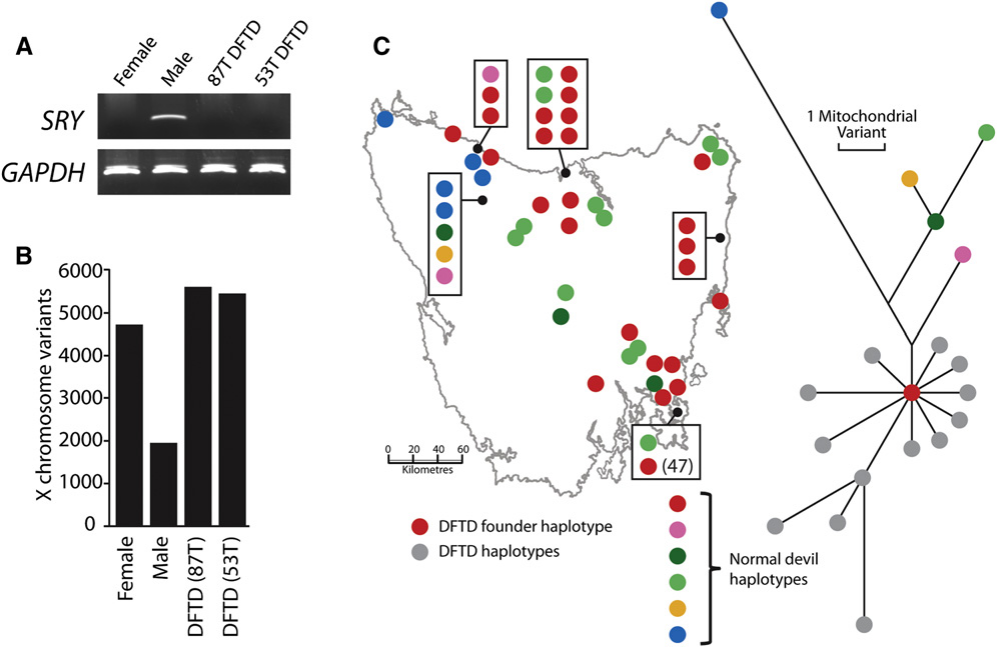
DFTD Origin (A) Y chromosome gene *SRY* is not detectable in DFTD using PCR. Primer sequences are available (Lachish et al., 2011; Murchison et al., 2010). (B) Number of X chromosome variants in female and male normal devil genomes and 87T and 53T DFTD genomes. Variants from a poorly assembled region at the end of chromosome X were excluded from this analysis. (C) Phylogenetic tree of devil mitochondrial variation. Each dot on the map indicates an individual devil and the color of the dot represents the mitochondrial haplotype for each devil. Each haplotype is also represented on the phylogenetic tree. DFTD mitochondrial haplotypes are indicated in gray; some DFTD tumors also had the haplotype represented by the red dot. See also Figure S3 for chromosome X copy number plots for 87T and 53T.

The gender of the founder devil is unknown. Like other marsupials, Tasmanian devils have X and Y sex chromosomes, and males are the heterogametic sex. Previous studies have indicated that neither of the sex chromosomes is cytogenetically identifiable in DFTD (Pearse and Swift, 2006). It is possible that the sex chromosomes initially present in the constitutional genome of the founder devil have been lost during DFTD carcinogenesis or that these chromosomes have been rearranged in the DFTD genome such that they are not cytogenetically identifiable. We first searched for the presence of the Y chromosome gene *SRY* in DFTD. As expected, the *SRY* gene could be amplified from the genome of a male devil but not from a female devil; however, our assays could not detect *SRY* in the DFTD genome (Figure 3A). We next searched for evidence of the X chromosome in the DFTD genome. Reverse chromosome painting experiments and copy number analysis of 87T indicated that the X chromosome is present in approximately two copies in this genome (Figure 2 and Figure S3). These are likely to be a homologous pair rather than recent duplicates, as the number of single-base substitution variants mapping to the X chromosome in the two DFTD genomes was comparable to the number of variants found on the X chromosome in the female normal devil genome and approximately double the number of X chromosome variants found in the male normal genome (Figure 3B). The data therefore suggest that the DFTD founder devil was a female.

DFTD was first observed in northeast Tasmania (Hawkins et al., 2006). To explore further the geographic origin of the founder devil we sequenced the mitochondrial genomes (excluding the control region) of 92 Tasmanian devils from 25 locations in Tasmania and constructed a phylogenetic tree based on their sequences (Figure 3C). We found evidence for six mitochondrial haplotypes among normal devils. Three of these had widespread distributions throughout Tasmania and three were confined to locations in the northwest of Tasmania, consistent with other studies (Miller et al., 2011). The 87T and 53T DFTD mitochondrial genomes were most closely related to one of the widespread devil haplotypes.

There is evidence of horizontal transfer of mitochondrial genomes between hosts and cancers in another transmissible cancer lineage, CTVT, which has led to multiple distinct clades of CTVT mitochondrial haplotypes (Rebbeck et al., 2011). To test whether horizontal transfer of mitochondria occurs in DFTD, we sequenced the mitochondrial genomes of 104 DFTD tumors and included their haplotypes on the phylogenetic tree (Figure 3C). All of the DFTD mitochondria were either identical to or apparently derived from a single devil haplotype, suggesting that they are clonally derived from the founder devil. These analyses suggest that mitochondrial horizontal transfer does not occur or is not widespread in DFTD, and indicate that the founder devil belonged to a haplogroup that is currently widespread throughout Tasmania.

DFTD was first observed in 1996 (Hawkins et al., 2006). However, we do not know the timing of the emergence of the DFTD clone. Given the overt and disfiguring symptoms of DFTD, as well as its dramatic recent effects on devil population size (Hawkins et al., 2006; Lachish et al., 2007), it seems unlikely that the disease remained undetected for a long period prior to 1996. Indeed, retrospective studies of devil skulls, preserved specimens and pelts collected between 1941 and 1989 revealed no evidence for DFTD prior to the 1990s (Loh et al., 2006a). However, it is possible that the current DFTD epidemic is the most recent manifestation of an ancient clone with a long history of coexistence with the Tasmanian devil population. Our mitochondrial genome analysis indicates that the founder devil's mitochondrial genome is identical to those found in many modern devils (Figure 3C). In addition, DFTD mitochondrial genomes are in most cases more closely related to the founder than to each other (Figure 3C). These observations are consistent with a recent origin for DFTD.

### Somatic Evolution of DFTD

Having identified genetic features and variants present in the constitutional genome of the DFTD founder devil, we next performed a detailed analysis of DFTD variants of somatic origin. Somatic variants are those that have arisen during the establishment and progression of DFTD as a clonal lineage. Analysis of somatic variants in two divergent DFTD lineages may provide insight into the mutational processes that have operated in DFTD as well as the genetic changes that have driven its growth.

**Figure 4:**
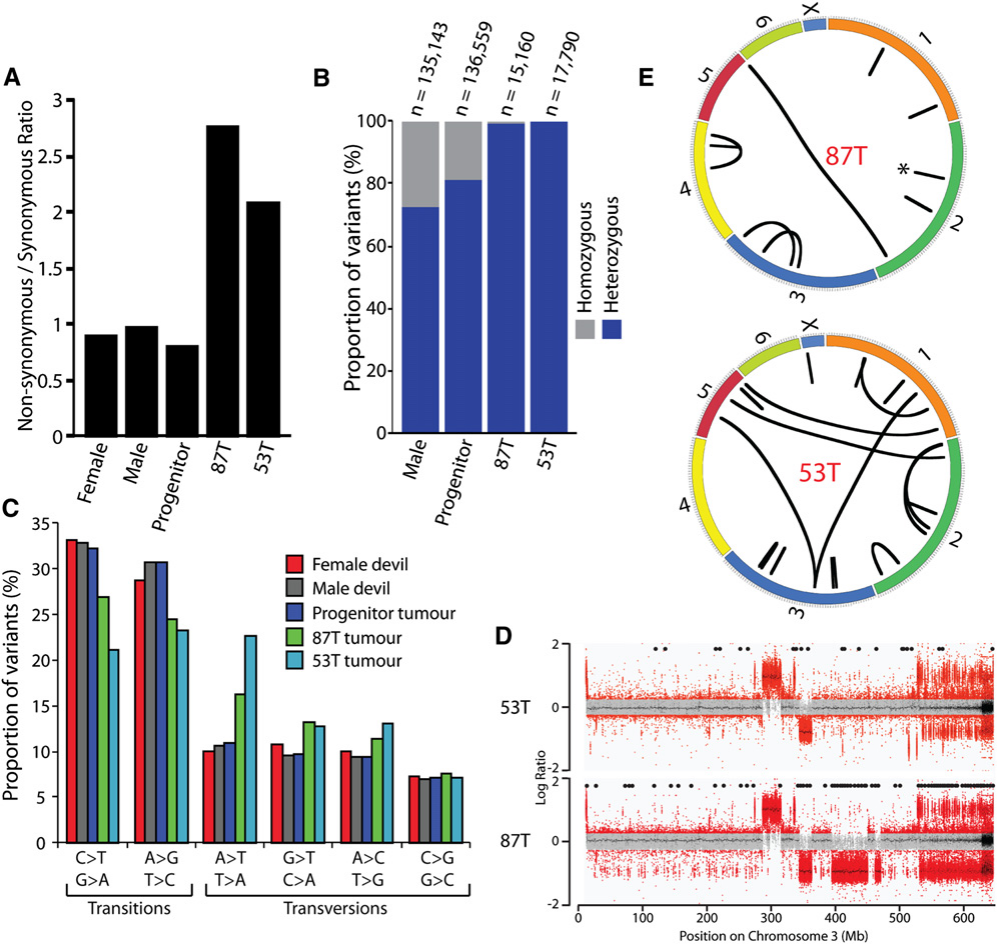
Somatic Evolution of DFTD (A) Nonsynonymous to synonymous ratios for variants occurring in genes in DFTD and in normal devil genomes and for variants inferred in the most recent common ancestor tumor of 87T and 53T (the progenitor). Only variants that were unique to the respective genomes were included in the analysis. Nonsynonymous gene variants in DFTD are listed in Table S4. (B) Heterozygosity for variants unique to the normal male genome, DFTD genomes and inferred in the most recent common ancestor tumor of 87T and 53T (the progenitor). (C) Mutation spectrum of single-base substitutions in DFTD and normal devil genomes. Only variants that were unique to the specified sample(s) were included in the analysis. The spectrum and ratios of the most recent common ancestor (progenitor) tumor (which includes the germline variants of the founder devil) were calculated using the common variants between 87T and 53T that were not present in the normal devil genomes. (D) Copy number analysis of Tasmanian devil chromosome 3 in 53T and 87T. Each dot represents the log_2_ ratio (that falls within the range −2 to +2) between the number of sequence reads in the tumor genome and the number of sequence reads in the female normal genome that align within a 2 kb genomic window. If p < 1 × 10^−5^, the dot is red; otherwise, dots are gray. Homozygous variants unique to either 53T or 87T are shown as black dots above the copy number plot. See Figure S3 for genome-wide comparison of 87T and 53T copy number. (E) Structural variants unique to 87T and 53T. Each chromosome is represented by a colored bar and black lines indicate either large-scale rearrangements (connecting lines) or small-scale rearrangements (single lines). Three 87T rearrangements that occurred close together on chromosome 2 are represented with a single bar (^∗^). See Table S3 for rearrangement coordinates. See also Figure S3 and Tables S3 and S4.

We cannot directly ascertain the set of somatic variants in DFTD because the founder devil died in obscurity in the Tasmanian bush more than a decade ago. However, we compiled a set of DFTD single-base substitutions enriched for somatic mutations by identifying variants that were present in one DFTD genome but absent in the other. We identified 15,160 single-base substitutions that were present in 87T but not 53T, and 17,790 that were in 53T but absent from 87T (Figure 2B). These variants could have arisen as somatic base substitution mutations. Alternatively, as we do not know the germline genotype of the DFTD founder devil, they could have been heterozygous germline variants that were lost in either of the two DFTD lineages. However, we established that most of these variants are likely to have arisen as somatic substitutions by demonstrating the absence of 15 out of 16 in the genomes of 110 normal devils. Moreover, the nonsynonymous to synonymous (NS/S) ratios for the 87T and 53T unique variants were 2.78 and 2.08 respectively, a range typical of somatic variants in human cancers and compatible with that expected from random mutagenesis (Figure 4A). By contrast, the NS/S ratios of germline devil single-nucleotide polymorphisms (SNPs) were 0.9 and 0.98 for the normal male and female devil genomes, respectively, similar to that of common SNPs in humans and indicative of substantial negative selection. Finally, as somatic mutations are likely to arise in the heterozygous state, the observation that variants unique to each DFTD lineage contain a high proportion of heterozygous variants provides further evidence for these sets being strongly enriched for somatic mutations (Figure 4B).

These estimates suggest that 87T and 53T have each acquired between 15,000 and 17,000 single-base substitution mutations since divergence from their most recent common ancestor tumor. We do not know how many somatic mutations were present in the DFTD lineage prior to 87T and 53T divergence. However, the observation that the total number of private variants inferred in the most recent common ancestor tumor (136,559) is comparable to the number of private variants in a normal male genome (135,134), as well as the NS/S ratio for these variants (0.8), suggests that the large majority of the private variants in the common ancestor tumor were of germline origin. This suggests that the prevalence of somatic substitution mutations in DFTD may not be substantially greater than 17,000. This is somewhat higher than the number of mutations observed in many human tumor types (approximately 5,000 per cancer genome) (Greenman et al., 2007). However, it is less than are found in many human melanomas and lung cancers, which are often the result of past mutagenic exposures, or in human cancers with mutator phenotypes due to DNA mismatch repair defects (Pleasance et al., 2010a, 2010b).

Cancers often have mutational processes that are different to those which operate in the germline. Comparison of the mutation spectra of Tasmanian devil germline variation to the sets of variants highly enriched for somatic mutations in 87T and 53T revealed that, as expected, devil germline SNPs were enriched for transitions (Figure 4C). However, we also observed elevated proportions of A:T → T:A, A:T → C:G, and G:C → T:A transversion mutations in DFTD (Figure 4C). This pattern was independently detectable in 87T and 53T since their divergence from their most recent common ancestor tumor, but was not detectable in the variants inferred in these two tumors' most recent common ancestor (Figure 4C). This suggests that this mutation profile is the result of an endogenous mutational process—for example, a defect in DNA repair—that was acquired before the divergence of the two lineages, or that it was caused by independent exposure of the two lineages to a carcinogenic environmental agent.

Copy number changes and structural rearrangements are commonly somatically acquired by cancer genomes. Although the majority of the copy number variants that we identified in 87T and 53T were common to both lineages (Figure S3), some copy number variants, including, for example, the hemizygous deletion on chromosome 3 in 87T, occurred in only one of the two tumors (Figure 4D). Such variants are likely to have arisen since the divergence of the 87T and 53T tumor lineages and have therefore been somatically acquired during DFTD evolution. We identified and validated 11 and 17 rearrangements that were specific to the 87T and 53T DFTD genomes, respectively (Figure 4E and Table S3). Thirteen of the 28 rearrangements unique to either 87T or 53T had between two and six bases of microhomology at the breakpoint region, indicating that DFTD may employ microhomology-mediated end joining as a repair process for double-stranded DNA breaks.

Most of the somatic variants that are present in DFTD are likely to be selectively neutral passenger mutations. However, a subset of somatic variants in the DFTD genome will be driver mutations that have provided selective advantage to the cancer during passage through its devil hosts. Three hundred twenty-four genes were predicted to contain nonsynonymous substitution and indel variants that were present in 87T and 53T but not in either of the normal devil genomes (Table S4). These included 313 genes with single-base substitutions and 11 genes with indels. A search for predicted nonsynonymous mutations in a set of 138 genes that are known to be mutated by single-base substitutions and indels in human cancers (Futreal et al., 2004) yielded heterozygous single-base substitutions in *RET* and *FANCD2* that were not present in either of the two normal genomes that we sequenced. Both mutations were predicted to cause single base substitution mutations that have not previously been described in cancer (Table S4).

Changes in the copy number of cancer genes and truncation or fusion of genes through rearrangements can also promote oncogenesis. Two genes, *MAST3* and a novel gene with similarity to *BTNL9*, were predicted to be homozygously deleted in DFTD. The functions of these two genes are not well understood, although the butyrophilin gene family, of which the *BTNL9*-like gene is a member, may be involved in immune modulation (Arnett et al., 2009; Stammers et al., 2000). Neither of the DFTD genomes contained any predicted regions of high-level amplification (Figure S3). We found several putative rearrangements involving genes, including a balanced translocation involving *PDGFA* (Table S4).

Although DFTD is not virally transmitted, it is possible that a virus may have contributed to DFTD pathogenesis. We searched for the presence of virus DNA in DFTD by aligning virus-derived DNA sequences contained in the RefSeq database with the assembled DFTD genomes as well as the normal devil genome assembly. We did not find evidence for exogenous viruses in the DFTD genome. However, it is possible that DFTD contains viral sequences that were not detectable using this method.

DFTD colonizes its devil hosts as an allogeneic graft. In order to investigate the mechanisms whereby DFTD evades host immune rejection, we searched for genetic variants in 25 genes involved in the antigen processing and presentation machinery (described by gene ontology IDs GO:0019885 and GO:0019882). Fifteen of these genes could be identified in the devil genome, and one gene, *NOD1*, had a predicted rearrangement that was predicted to be present in both DFTD genomes but absent from the normal Tasmanian devil genomes (Table S4). Further analysis and annotation of immune genes in the Tasmanian devil genome will be required to elucidate the genetic mechanisms of DFTD immune evasion.

### Divergence and Clonal Dynamics of DFTD Lineages

We have described the somatic changes that have occurred in two DFTD cancers, 87T and 53T, collected in the Forestier Peninsula in the southeast of Tasmania and Narawntapu National Park on the north coast of Tasmania, respectively, since divergence from their most recent common ancestor tumor. Observational epidemiological studies have indicated that DFTD first arrived in the Forestier Peninsula in 2004. The first DFTD case observed in Narawntapu National Park was in 2007. However, we do not know the routes that were followed by these lineages across Tasmania, nor do we have any information about the clonal dynamics of DFTD disease spread. We investigated whether DFTD progression into new territories is characterized by linear colonization and occupation, or rather by repeated waves of lineage replacement.

**Figure 5:**
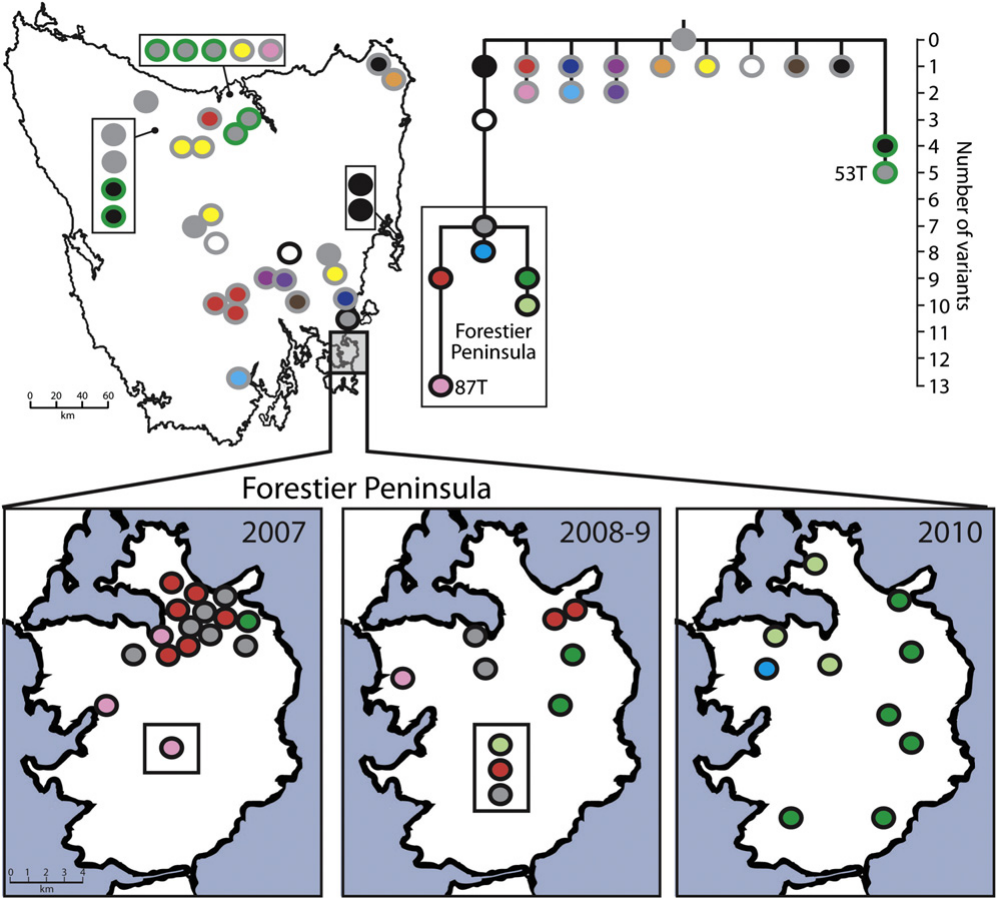
DFTD Clonal Dynamics Phylogenetic tree summarizing genetic variation found in 104 DFTD tumors collected from 69 Tasmanian devils. The tree was constructed using both nuclear and mitochondrial variants and branch length represents the number of variants (either nuclear or mitochondrial) that distinguish each tumor type from the most likely ancestral tumor type (solid gray). Trapping locations for devils captured with DFTD are indicated either on the map of Tasmania (top) or on the map of the Forestier Peninsula (bottom), with colors indicating the genetic subgroup to which each animal's tumor(s) belongs. Four Forestier Peninsula tumors for which trapping location data were not available are indicated in boxes. The six cases in which a single devil had multiple tumors with more than one genotype are represented on the map with just one genotype. See also Figure S4 for further details about devils with multiple tumors and Table S5 for genome coordinates for variants.

The evolutionary dynamics of the DFTD clone during its expansion across Tasmania can be traced by analysis of the observed patterns of somatic mutation. We collected 104 DFTD tumors from 69 Tasmanian devils captured in several locations throughout Tasmania between 2004 and 2010 (Figure 5). We genotyped this set of tumors for 16 variants that we had previously identified either in 87T or 53T but not in both tumors and thus are likely to be somatic (Table S5). In addition, we analyzed the mitochondrial genomes (excluding the control region) from the entire set of DFTD tumors, leading to the identification of 21 somatic mitochondrial DFTD variants (Table S5). These experiments revealed differences in the population of DFTD in different regions of Tasmania.

The observation that all of the tumors in the isolated Forestier Peninsula cluster into a single lineage suggests that this tumor population was founded by a single subclone of DFTD, precursors of which are located on the east coast of Tasmania (Figure 5). Divergence within this lineage after its introduction has given rise to a number of tumor subclones found only within the Forestier Peninsula. One of these lineages (illustrated in Figure 5 with a green dot, black outline) appears to have increased in frequency between 2007 and 2010 in a manner resembling a selective sweep (Figure 5, lower panel). These fluctuations in the dominant tumor type could be due to selection, or they could alternatively be due to simple neutral processes.

In contrast to the Forestier Peninsula, the mainland Tasmanian DFTD population shows the emergence and simultaneous maintenance of several distinct tumor subclones (Figure 5). The tumor lineage to which 53T belongs appears to be a dominant clone in the north and northwest (Figure 5, dots with green outline). Several tumors were found to have unique patterns of variation. For example, each of the two tumors that we sampled from northeastern Tasmania, the location where DFTD was first observed in 1996, had their own individual patterns of variation (Figure 5, orange and black dots, gray outline), suggesting that tumor diversity may be greater in this region, perhaps reflecting its status as the possible origin of DFTD (Hawkins et al., 2006).

### DFTD Diversity within Individual Hosts

We collected two or more DFTD tumors from 20 individual devils in our set. In some cases the additional tumors were facial or oral, and in others they were in submandibular lymph nodes and internal organs. Genotyping was unable to distinguish between multiple tumors derived from the same host in 14 of the 20 cases, suggesting that most additional tumors are metastases of primary tumors originating from a single DFTD bite.

**Figure S4:**
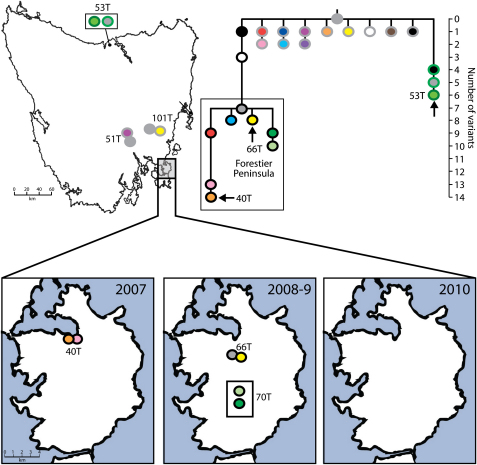
Analysis of Multiple DFTD Tumors from the Same Host, Related to Figure 5 Six Tasmanian devils (Devil IDs 40, 51, 53, 66, 70 and 101) were identified with two more or more DFTD tumors with different genotypes. In three of these cases (Devils 40, 53, 66), one of the tumors had a new genotype that had not been identified in any other animal (arrows on phylogenetic tree). In the remaining three cases (Devils 51, 70 and 101) the second genotype could be identified in other animals. The locations where the six devils with non-matching genotypes were trapped are indicated on the map. All DFTD cases were confirmed by genotyping, and any tumor with unacceptably high levels of contaminating host DNA was excluded from the analysis. The corresponding genotypes for each devil are listed in Table S5.

There were six cases, however, in which an individual animal had tumors with two different genotypes (Figure S4). In three of these cases, both genotypes were found in tumors in other animals, indicating that the two tumors were probably derived from separate DFTD bites. This suggests that prior exposure to DFTD does not protect devils from subsequent DFTD inoculations. However, in each of the remaining three cases, one of the two genotypes was not found in a tumor in any of the other animals that we sampled. In these instances, the two genotypes differed only by a single variant, and it is possible that the novel genotypes may have arisen as new variants in these animals (Figure S4).

## Discussion

Cancer genomes bear imprints of carcinogenic exposures, endogenous DNA repair processes and selective pressures to which the clone has been subject. DFTD has existed as a malignant clonal lineage for at least 15 years by repeated subcloning through the Tasmanian devil population. Our analysis of the genomes of two geographically distant DFTD subclones has indicated that DFTD is continuing to acquire new variations in its karyotype, genomic copy number and DNA sequence. Despite evidence for ongoing somatic change in the DFTD lineage, the overall level of mutation that has been accrued by two DFTD lineages since divergence from their most recent common ancestor tumor is comparable with the number of changes that are observed within some human cancers (Pleasance et al., 2010a, 2010b). This is perhaps surprising, given that DFTD has probably undergone a greater number of mitoses than most human cancers. It indicates, however, that DFTD is a relatively stable lineage and that a high level of genomic instability has not been required for the cancer to become transmissible.

Analysis of the genetic diversity of DFTD subclones throughout mainland Tasmania suggests that the evolution of DFTD has been characterized by linear radiation of DFTD subtypes from their common origin. Geographical analysis of DFTD lineage diversity indicates a wide distribution of variant DFTD subclones as well as local coexistence of different subclones, sometimes even within a single host. Our analysis identified a DFTD founder population on an isolated peninsula. Divergence within this lineage has led to the appearance of several DFTD subtypes, one of which has recently become dominant in a manner resembling a selective sweep. Future genomic analysis of hundreds of DFTD genomes will provide further insight into the diversity and evolution of DFTD, and may perhaps help to predict the future trajectory of this clone and its impact on the devil population.

The transfer of cancer cells between individuals is normally prevented both by physical barriers and by the action of the immune system. The ability of transmissible cancers to circumvent these obstacles demonstrates the potential of cancer cells to become parasitic clonal lineages. DFTD and CTVT are the only two known naturally occurring transmissible clonal lineages. Our studies have highlighted similarities and differences between the two lineages. Previous reports have indicated that the most recent common ancestor of today's globally distributed CTVT clones existed between 47 and 2,000 years ago (Murgia et al., 2006; Rebbeck et al., 2009). DFTD, however, is probably not more than 20 years old. CTVT has been observed to periodically take up mitochondria from its host by horizontal transfer (Rebbeck et al., 2011); in contrast, we do not find any evidence for this phenomenon in DFTD. Interestingly, it has been proposed that many modern CTVT tumors represent the most recent global sweep of a subclone of the disease (Rebbeck et al., 2009). We observed a similar sweep of DFTD tumors on the Forestier Peninsula. Both CTVT and DFTD continue to acquire new copy number variations (Thomas et al., 2009). Future analysis of both the DFTD and CTVT lineages and their hosts will help to determine the common and unique features of these two cancers and will perhaps reveal common genetic changes that favor the outgrowth and progression of clonally transmissible cancers.

Although there are no known naturally occurring transmissible cancers that affect humans, there are rare reports of cancer transmission between two or more humans. These involve accidental transfer of cancer cells through organ transplantation or during surgical procedures, deliberate transfer of cancer cells between humans for experimental purposes, or transfer of cancer cells in utero (Gandhi and Strong, 2007; Gärtner et al., 1996; Moore et al., 1957; Tolar and Neglia, 2003). Further comparative studies of transmissible cancer genomes may indicate the mechanisms that permit cancer transmission between individuals.

Cancer is an inevitable outcome of the potential of cells to reproduce and to adapt to their environment; their environment is usually limited to a single host, but cancers can sometimes escape from their hosts and become parasitic clonal lineages. Here we have described a whole-genome analysis of such a cancer, and our studies have provided insights into the genetic identity of the individual that founded the DFTD clone, as well as patterns of ongoing DFTD somatic evolution and clonal dynamics. This work will enable more detailed studies of the structure and history of the Tasmanian devil population and its response to the DFTD epidemic. Understanding the interaction between the genomes of DFTD and its host and the identification of patterns of disease spread and host response may provide information that will assist with the conservation of the Tasmanian devil.

## Experimental Procedures

### Whole-Genome Sequencing, Assembly, and Annotation

DNA from a female Tasmanian devil fibroblast cell line (with trisomy 6) and from two DFTD cell lines (87T and 53T) was extracted and used to prepare short insert libraries and mate pair libraries with insert sizes from 3–10 kb (fibroblast cell line only) for paired end sequencing as previously described (Bentley et al., 2008). Short-insert library sequencing with 100 bp paired-end reads was performed on an Illumina HiSeq2000 instrument and mate-pair library sequencing with 50 bp paired-end reads was performed on an Illumina GA2 instrument. In addition, short-insert libraries were constructed from DNA extracted from the liver of a male Tasmanian devil. The library was sequenced on an Illumina Genome Analyzer IIx machine with 108 bp reads. Sequencing, raw data processing, and quality-control checks were performed as previously described (Bentley et al., 2008).

The genome of the female devil was assembled with the Phusion2 Assembly Pipeline (Mullikin and Ning, 2003). In brief, paired-end sequence reads were processed to generate kmer words (k = 61). K-tuples were merged and sorted into a table, and shared kmer words were linked in a relation matrix. Small read clusters with ∼100,000 reads were used to generate contigs with Phrap (http://www.phrap.com/). RPono, a package in the Phusion2 pipeline, was then used to build supercontigs with mate-pair sequences. Genome size was estimated using kmer frequency information, flow karyotype analysis and nuclear DNA content analysis (see the Extended Experimental Procedures, Figure S1, and Table S1). The genome was annotated with the Ensembl Genebuild Pipeline (Curwen et al., 2004; Potter et al., 2004). Transcriptome sequencing of pooled RNA from 12 devil tissues was used to assist with annotation (more details are in the Extended Experimental Procedures).

### Chromosome Assignment

Each of the seven Tasmanian devil chromosomes was individually sorted from the female devil fibroblast cell line with a flow cytometer. Fifty thousand copies of each devil chromosome were collected, amplified, and sequenced on two lanes of an Illumina Genome Analyzer IIx instrument with 100 bp paired-end reads. Alignment of chromosome-derived reads with contigs was used to assign contigs to chromosomes and to correct assembly errors. We assigned 35,534 supercontigs (99%) to individual chromosomes (Table S2). Supercontigs were ordered on chromosomes using conservation with opossum, and supercontigs that could not be assigned to a chromosome were assigned to “ChrU.” Chromosome assignment was validated with fluorescence in situ hybridization. Sorted chromosomes were also used as probes for chromosome painting (Extended Experimental Procedures).

### Variant Analysis

Reads were aligned with the reference genome using BWA (Li and Durbin, 2009). Single-base substitutions (Figure 2B) were called using SAMtools and filtered by coverage (minimum 10, maximum 150), read quality (minimum quality, 30), mapping quality (minimum quality, 30), base quality (minimum quality, 30), and end of contig (minimum distance to end of contig, 500 bp). Indels (Figure 2B) were called using CASAVA with parameters Q(snp) ≥ 30 and Q(max_gtype) ≥ 5. One hundred eleven of 117 (95%) single-base substitutions and 119 of 124 (96%) indels, randomly selected, were confirmed with capillary sequencing. Variants from each genome were compared and subtracted to identify the set of variants that were unique to each genome.

Structural rearrangements were identified as previously described (Pleasance et al., 2010a). In brief, read pairs were aligned with the draft Tasmanian devil assembly with BWA (Li and Durbin, 2009). Discordant pairs that mapped with an unexpected insert distance or orientation or to different supercontigs were identified and clustered to form regions of interest. We discarded groups which did not have at least seven reads of mapping quality ≥30 supporting the variant, as well as all reads that were within 500 bp of the end of a contig. Structural variants were filtered for those that were specific to individual samples and a subset were validated with PCR, gel electrophoresis, and sequencing from both ends with an ABI 3730xl DNA analyzer. Mitochondrial genomes (excluding the control region) were sequenced with the capillary platform, and variants were called with NovoSNP (Weckx et al., 2005). Copy number variants were identified using the DNAcopy package (Olshen et al., 2004) with a nonoverlapping window of 2,000 bp. A subset of copy number variants were validated with quantitative real-time PCR.

### Tasmanian Devil Samples

Tissue samples were collected from wild and captive Tasmanian devils under research authorities 33/2004-2005 and 24/2006-2008 (extended) issued by the Tasmanian Department of Primary Industries, Water, and the Environment. The research was reviewed by the Wellcome Trust Sanger Institute Animal Ethics Committee.

Extended Experimental Procedures **Tasmanian Devil Samples and Cell Lines**

Tasmanian devil (*Sarcophilus harrisii*) samples were collected from captive animals or from wild animals either in the field, or at postmortems undertaken at the Tasmanian Department of Primary Industries Animal Health Laboratories. Cell lines were established from two DFTD tumors and from the skin of a normal captive-bred five-year-old female Tasmanian devil. The female fibroblast cell line developed trisomy 6 during cell culture.

**Tasmanian Devil Genome Sequencing**

DNA from a female Tasmanian devil fibroblast cell line and from two DFTD cell lines (87T and 53T) was extracted and used to prepare short insert libraries (fibroblast and DFTD cell lines) and mate pair libraries with insert sizes from 3–10 kb (fibroblast cell line only) for paired end sequencing as previously described (Bentley et al., 2008), with the following modifications; libraries were generated with TruSeq Adapters and a single cycle of PCR. Short insert library sequencing with 100 bp paired end reads was performed on an Illumina HiSeq2000 instrument and mate pair library sequencing with 50 bp paired end reads was performed on an Illumina GA2 instrument. In addition, short insert libraries were constructed from DNA extracted from the liver of a male Tasmanian devil; the library was generated with TruSeq Adapters and 10 cycles of PCR. The library was sequenced on an Illumina Genome Analyzer IIx machine with 108 bp reads. Sequencing, raw sequencing processing and quality control checks were performed as previously described (Bentley et al., 2008).

**Tasmanian Devil Genome Assembly**

We developed the Phusion2 genome assembly pipeline to assemble large eukaryotic genomes using Illumina short sequence reads. Read files from individual lanes were processed to generate kmer words at a given size (k = 61). K-tuples were then merged and sorted into a table so that kmer words shared by different reads could be linked. A relation matrix was used to record the shared kmer words among all the reads. Setting a minimum threshold of shared k-tuples, short reads can then be clustered into groups using kmer sharing information in the relational matrix. After obtaining small read clusters with a controllable size (∼100,000 reads), we used Phrap (http://www.phrap.com/) to generate contigs.

For the normal female Tasmanian devil genome, we generated 2.87 × 10^9^ 100 bp reads. Using k = 61, the kmer frequency peaks at 25. Any kmer word occurring more than 35 times was not used in further analysis, thus removing the repetitive reads. Since single end reads are combined into “pairs,” the gaps due to missing single repetitive reads are filled by paired partners. After read clustering, we obtained 390,842 read groups containing 1.022 × 10^9^ read pairs. Using Phrap, we generated an assembly with 2.93 Gb of assembled bases and an N50 of 20 kb.

Incorporating mate pair sequence data (194 × 10^6^ paired reads of 2x50 bp with insert sizes from 3 to 10 kb) allowed us to obtain a scaffold assembly. The PCR duplication rate of the mate pair sequence reads was very low (<5%). We removed chimeric read pairs by excluding read pairs if one or two ends were found in the middle of the contig, but the edge length was larger than the insert size. To minimize the effect of read pairs overlapping biotin junctions (the circularization junction point that is introduced during mate pair library construction) we only used those pairs with full-length genome alignment. We then used RPono, a package in the Phusion2 pipeline, to build supercontigs. At the end of this process, we had an assembly of 3.15 Gb with an N50 of 839 kb. To further improve our assembly, we aligned our supercontigs with the opossum genome assembly (Monodelphis5.0); this yielded our draft assembly with a total of 3.17 Gb assembled bases and with an N50 of 1.84 megabases (Mb) (Table 1). This version of the genome assembly (Tasmanian devil genome assembly version 7.1) was used for all of the analyses described in this paper (Table 1).

In addition, we performed de novo assemblies of the two DFTD genomes, 87T and 53T, using similar methods. These assemblies have been deposited in the GenBank/DDBJ/EMBL database.

The Tasmanian devil mitochondrial genome was amplified from DNA extracted from the female Tasmanian devil fibroblast cell line. Amplicons were sequenced from both ends with an ABI 3730xl DNA Analyzer and manually assembled. The control loop region could not be completely sequenced due to its repetitive structure.

**Genome Size Estimation Using Kmer Frequency**

We define the genome size as the total number of effective kmer words divided by the kmer depth or the kmer occurrence number at the peak kmer frequency *D_p_*:

(S1) $$G_{s}=\frac{\left(K_{n}-K_{s}\right)}{D_{p}}.$$

Here *K_n_* is the total number of kmer words and *K_s_* is the number of single or unique kmer words. The peak depth values for the female Tasmanian devil genome and DFTD cell lines 87T and 53T are *N_p_* = 25, 18 and 27 respectively. With *K_n_* = 81.99 × 10^9^, 68.75 × 10^9^ and 91.20 × 10^9^ and the numbers of single occurrence kmer words are *K_s_* = 6.249 × 10^9^, 13.656 × 10^9^ and 9.135 × 10^9^, we estimate the genome size to be *G_s_* = 3.03 × 10^9^, 3.06 × 10^9^ and 3.04 × 10^9^ respectively (Table S1).

**Flow Karyotype Analysis of Tasmanian Devil DNA Content and Genome Size**

To estimate the genome and chromosome size of the Tasmanian devil by flow karyotype analysis, normal Tasmanian devil fibroblast chromosome suspensions were compared to chromosome suspensions from human lymphoblastoid cell lines, both prepared as previously described (Gribble et al., 2009). Briefly, Tasmanian devil and human chromosome suspensions as well as human-devil mixed chromosome suspensions were stained overnight with Hoechst 33258 (HO) and Chromomycin A3 (CA3) and analyzed on a flow cytometer as described previously (Gribble et al., 2009). A total of 50,000 to 200,000 events were acquired for each chromosome preparation and displayed on a bivariate plot of HO versus CA3 fluorescence. Data collected from the experiments were analyzed using the Summit analysis software (Beckman Coulter).

In order to estimate the Tasmanian devil chromosomal DNA content and genome size, we made use of a technique described by Trask and Schmitz (Schmitz et al., 1992; Trask et al., 1989) using a ‘DNA line’ in the human flow karyotype as a standard to estimate the Tasmanian devil chromosome DNA size. This approach involved acquiring data from a mixed human-Tasmanian devil chromosome suspension and calculating the mean HO and CA3 fluorescence intensity for a few selected human chromosomes and each of the Tasmanian devil chromosome peaks along the human DNA line. The human DNA line is a projection through the origin and the peak of a normal human chromosome 4 at an angle (α) to the *x* axis which in this case was 50°. DNA values for chromosome peaks were computed using the formula described by Trask (Trask et al., 1989),

(S2) $${\text{D}}_{n}={\text{HO}}_{n}\;\text{X}\;\sin\;\alpha +\text{CA}3_{n}\;\text{X}\;\cos\;\alpha .$$

The chromosomal DNA content for each of the Tasmanian devil chromosome peaks were determined by linear regression using the measured human D*_n_* value for selected chromosomes and the estimated human chromosomal DNA content (Trask et al., 1989). The genome size of the Tasmanian devil was obtained through the summation of the chromosomal DNA content of all the measured chromosome peaks (Table S1).

**DNA Content Analysis of Cells Using Propidium Iodide**

The genome size (DNA content) of Tasmanian devil cells was estimated by DNA index analysis. DNA cell suspensions were prepared from lysed whole blood isolated from four different Tasmanian devils. Four different human lymphoblastoid cell lines were analyzed together using propidium iodide (PI) staining. Commercially available chicken erythrocytes nuclei (CEN) of known DNA content acted as an internal reference standard.

The DNA cell samples from fixed lysed whole blood of Tasmanian devil and human lymphoblastoid cell lines were stained with PI solution before analyzing on a flow cytometer (Darzynkiewicz and Juan, 1997). A total of 10,000 events were acquired per DNA cell sample. The cells were gated on PI fluorescence area versus PI fluorescence width to discriminate any doublets and clumps. The gated events were displayed on a histogram plot of PI fluorescence area (Figure S1). Data collected from the experiments were analyzed using the Summit analysis software (Beckman Coulter).

The Tasmanian devil genome size was estimated as follows. An aliquot of CEN was mixed with 1 × 10^6^ Tasmanian devil cells at a ratio of 1:20. Flow cytometric data were acquired from the mixed CEN-Tasmanian devil DNA cell suspension and the DNA Index (DI) was measured by calculating the mean PI fluorescence intensity of each peak and finding the ratio of the mean value of the Tasmanian devil peak to CEN as shown in Figure S1. The genome size of the Tasmanian devil was calculated using the formula

Genomesize(Mbp)=CENgenomesize(pg)×(DI:TDmeanpeak/CENmeanpeak)×(978Mbp/pg),

with CEN genome size = 1.25 pg.

The average genome size of Tasmanian devil was computed and is shown in Table S1.

Human cells were used as a validation of the DNA index analysis (Figure S1). The genome size for human was calculated using the formulas above and compared to the value obtained from current sequence estimation. A comparison of the Tasmanian devil and human genome sizes is shown in Figure S1.

**In Silico Chromosome Assignment**

To determine the chromosomal locations of our assembled supercontigs, each of the seven Tasmanian devil chromosomes was individually sorted from the female devil fibroblast cell line using a flow cytometer. 50,000 copies of each devil chromosome were collected, amplified, and sequenced. The chromosomes were ∼90% pure, based on previous experience; however, it is possible that there was some degree of contamination between chromosomes with similar positions on the flow karyotype (e.g., chromosomes 2 and 3). Chromosomes were amplified using the Genomiphi Whole Genome Amplification Kit (GE Healthcare). Individual libraries were prepared for the flow sorted chromosomes with average insert sizes of ∼300 bp. Construction of paired end libraries was essentially as described previously (Quail et al., 2008). DNA fragmentation was targeted to 500-700bp model peak (AFA; Covaris settings: duty cycle 20%, intensity 5, cycle burst 200 for 30 s). The NEBNext DNA Sample Prep Reagent Set 1 was used with incubation times for end repair and A-tail stages of one hour and a two hour ligation step, each step was followed by column purification using QIAquick spin columns (QIAGEN). A single agarose gel size selection step was performed (selecting DNA from 500-800bp) prior to PCR amplification of 5-8 cycles depending on input DNA (estimated using the Agilant 2100 Bioanalyser). The PCR product was cleaned using 0.6 vols SPRI beads (Agencourt) and the concentration of sequencable products estimated by qPCR (using the KAPA Library Quantification Kit).

Each chromosome library was sequenced on two lanes of an Illumina Genome Analyzer IIx instrument with 100 bp paired end reads with read coverage from 22-95x depending on the chromosome size. We first aligned all of the flow-sorted chromosome reads with the assembled contigs. We calculated the total number of mapped reads and the numbers of mapped reads from each chromosome library for each contig. Since the sizes of chromosome are different, we introduced an effective number of mapped reads for each contig:

(S3) $$N_{i,e}=\left(\frac{C_{i,s}}{N_{i,c}}\right)N_{i};\;\left(i=1,2,3,4,5,6,7\right).$$

Here *C_i,s_* is the size of chromosome *i*; *N_i,c_* is the number of raw flow-sorted chromosome sequence reads aligning to chromosome *i*; *N_i_* is the number of mapped reads from flow sorted chromosome sequence library *i*. For a given contig, we first calculated the maximum and the second maximum values of the effective mapping numbers: *N_m,e_* and *N_m-1,e_*. If the ratio of *N_m-1,e_* / *N_m,e_* was less than 0.4, this contig was assigned to the chromosome from which the effective number of mapped reads had the maximum value. If the ratio was larger than 0.4, we sorted the mapping coordinates along the contig and divided the mapped reads into blocks of 20 reads. For these small units, we examined blocks one by one for a transition from one chromosome library to another; such a transition is strong evidence for misassembly. This method allowed us to detect and correct 2,827 misassembled contigs.

Once we had an assembly in which the majority of contigs had been assigned to chromosomes and potential assembly errors had been corrected, we produced supercontigs using mate pair sequence reads and synteny with opossum. In these two steps, small unassigned contigs could be assigned to a chromosome together with other assigned contigs within one supercontig. Using this method we were able to assign 35,534 supercontigs (99%) to individual chromosomes. Supercontigs that could be assigned to a chromosome but could not be aligned with the opossum assembly were placed at the end of each chromosome assembly. Supercontigs that could not be assigned to a chromosome were assigned to “ChrU.”

**Transcriptome Sequencing**

Transcriptome sequencing was performed on cDNA libraries constructed from pooled RNA from 12 devil tissues (heart, liver, skin, spleen, testis, brain, kidney, lung, bone marrow, pancreas, adrenal gland and salivary gland). Three transcriptome libraries were prepared (total RNA library, mRNA library and mRNA-UDG library). Brief methods used for the preparation of each library, and details of the sequencing and analysis are provided below.

**Library Preparation**

**Ligation TotalRNA-Seq Library**

The totalRNA library was constructed Illumina's modified “directional mRNA-Seq Sample Preparation” protocol. Briefly, 100 ng of total RNA was fragmented with divalent cations under elevated temperature. The ends of the fragmented RNA were modified with polynucleotide kinase for a 5′ mono phosphate group and a 3′ hydroxyl group. A preadenylated oligo and a RNA oligo were ligated sequentially to the 3′end and 5′end of the RNA respectively. Adaptor ligated RNA was reverse transcribed and amplified with 15 cycles of PCR. DSN rRNA depletion is carried out following Illumina's “DSN normalization sample prep application note.” Briefly, 100 ng of amplified PCR products were hybridized in 1x hybridization buffer (50 mM HEPES, 0.5 M NaCl) at 68°C for 5 hr. 2U of DSN enzyme was used at 68c for 25min to digest double stranded DNA, and the remaining single stranded molecules are amplified with 12 cycles of PCR.

**mRNA-Seq Library**

The mRNA-seq library was constructed with the Illumina truseq RNA sample preparation protocol. Briefly, poly A+ RNA was purified from 100 ng of totalRNA with oligo-dT beads. Purified RNA was fragmented with divalent cations under elevated temperature. cDNA was synthesized with random hexamers. Double stranded cDNA was end repaired, an A base was added, and the product was ligated to Illumina PE adaptors. Adaptor ligated cDNA was amplified with 15 cycles of PCR. DSN cDNA normalization was carried out according to modified Illumina “DSN normalization sample prep application note.”

**mRNA-Seq-UDG Library**

The mRNA-seq library was constructed with a modified Illumina truseq RNA sample prep protocol. Briefly, poly A+ RNA was purified from 100 ng of totalRNA with oligo-dT beads. Purified RNA was fragmentated with divalent cations under elevated temperature. cDNA was synthesized with random hexamers. dUTP was incorporated during second strand cDNA synthesis. Double stranded cDNA was end repaired, an A base was added, and ligated to Illumina PE adaptors. DSN cDNA normalization was carried out according to the modified Illumina “DSN normalization sample prep application note.”

**Sequencing and Analysis**

We sequenced 863,429,107 reads in total on the Illumina platform (675 × 10^6^ pairs with 100/35bp read length, 188 × 10^6^ unpaired reads with 150 bp read length). About 40% of the reads were sequenced from a stranded RNA protocol. To test the gene coverage of our draft genome, we performed an independent de novo assembly of the paired RNA-seq reads using T-IDBA (Peng et al., 2011). We assembled 470,729 transcripts in 239,560 Mb. The RNaseq de-novo assembly had an N50 of 687 bp (94,029 contigs) and an N80 of 314 bp (250,268 contigs). The largest transcript had a size of 83,452 bp. We aligned them to the genome using BLAST (Altschul et al., 1990). 95.3% of the transcripts could be aligned with 0.05% mismatches and 0.01% gaps to the assembly which indicates that our genome assembly has coverage for the majority of coding sequences in the devil genome.

**Gene Annotation**

Tasmanian devil gene annotation was performed using the Ensembl Genebuild pipeline (Curwen et al., 2004; Potter et al., 2004), summarized below.

**1. Raw Compute Stage: Searching for Sequence Patterns, Aligning Proteins and cDNAs to the Genome**

The annotation process of the Tasmanian devil assembly began with the “raw compute” stage whereby the genomic sequence was screened for sequence patterns including repeats using RepeatMasker (version 3.2.8) (Smit et al., 1996–2010), Dust (Morgulis et al., 2006) and TRF (Benson, 1999). RepeatMasker and Dust combined masked 49.0% of the devil genome. Transcription start sites were predicted using Eponine-scan (Davuluri et al., 2001; Down and Hubbard, 2002). CpG islands and tRNAs were also predicted (Lowe and Eddy, 1997).

Genscan (Burge and Karlin, 1997) was run across repeat masked sequence and the results were used as input for UniProt (Goujon et al., 2010), UniGene (Sayers et al., 2010), and vertebrate RNA alignments by WU-BLAST (Altschul et al., 1990). (Passing only Genscan results to BLAST is an effective way of reducing the search space and therefore the computational resources required). This resulted in 269,535 UniProt, 309,553 UniGene and 284,670 Vertebrate RNA sequences aligning to the genome.

**2. Targeted Stage: Generating Coding Models from Devil Evidence**

Devil protein sequences were downloaded from public databases (UniProt SwissProt/TrEMBL and GenBank) and filtered to remove sequences based on predictions. The devil sequences were mapped to the genome using Pmatch (Slater and Birney, 2005). Models of the coding sequence (CDS) were produced from the proteins using Genewise (Birney et al., 2004). Two sets of models were produced, one with only consensus splice sites and one where non-consensus splices were allowed; where a single protein sequence had generated two different coding models at the same locus, the BestTargeted module was used to select the coding model that most closely matched the source protein to take through to the next stage of the gene annotation process. The generation of transcript models using devil-specific data is referred to as the “Targeted stage.” This stage resulted in 215 of 281 devil proteins used to build 215 coding models.

**3. cDNA and EST Alignment**

Devil cDNAs were downloaded from GenBank, clipped to remove polyA tails, and aligned to the genome using Exonerate (Birney et al., 2004). Of these, 21 of 27 devil cDNAs aligned with a cut-off of 90% coverage and 97% identity.

**4. Similarity Stage: Generating Additional Coding Models Using Proteins from Related Species**

Due to the paucity of devil specific protein and cDNA evidence, the majority of the gene models were based on proteins from other species. UniProt alignments from the Raw Compute step were filtered to favor proteins classed by UniProt's Protein Existence (PE) classification level 1 and 2. Proteins from other PE levels were used where no other evidence was available; similarly, mammalian proteins were favored over non-mammalian. WU-BLAST was rerun for these sequences and the results were passed to Genewise to build coding models. The generation of transcript models using data from related species is referred to as the “Similarity stage.” This stage resulted in 109,194 coding models.

**5. Filtering Coding Models**

Coding models from the Similarity stage were filtered using modules such as TranscriptConsensus, RNA-Seq spliced alignments supporting introns were used to help filter the set. 61,937 models were rejected as a result of filtering.

**6. Addition of RNA-Seq Models**

The largest set of devil specific evidence was from Illumina paired end RNASeq, this was used where appropriate to help inform our gene annotation. A set of 1.6 × 10^9^ reads was aligned to the genome using BWA (Li and Durbin, 2009). The Ensembl RNA-Seq pipeline was used to process the BWA alignments and create a further 86 × 10^6^ split read alignments using Exonerate (Slater and Birney, 2005). The split reads and the processed BWA alignments were combined to produce 41,011 transcript models in total; one transcript per locus. The predicted open reading frames were compared to Uniprot Protein Existence (PE) classification level 1 and 2 proteins using WU-BLAST, models with no BLAST alignment or poorly scoring BLAST alignments were discarded. The resulting models were added into the gene set where they produced a novel model or splice variant, in total 5,663 models were added.

**7. Pseudogenes, Protein Annotation, Non-coding Genes, Cross Referencing, Stable Identifiers**

The gene set was screened for potential pseudogenes. Before public release the transcripts and translations were given cross references to external databases, while translations were searched for domains/signatures of interest and labeled where appropriate. Stable Identifiers were assigned to each gene, transcript, exon and translation. (When annotating a species for the first time, these identifiers are auto-generated. In all subsequent annotations the stable identifiers are propagated based on comparison of the new gene set to the previous gene set.)

Small structured non-coding genes were added using annotations taken from RFAM (Griffiths-Jones et al., 2003) and miRBase (Griffiths-Jones et al., 2006). In addition, devil miRNAs were identified and annotated as described (Murchison et al., 2008) by aligning small RNA reads that were sequenced in a previous study (Murchison et al., 2010).

The final gene set consists of 18,775 protein coding genes containing 22,391 transcripts, 178 pseudogenes, 1,446 ncRNAs including 363 miRNAs.

**8. Cancer and Immune Gene Annotation**

We specifically searched for and annotated sets of genes with known causative roles in cancer (Futreal et al., 2004) or with known involvement in antigen processing and display in the immune system (described by Gene Ontology IDs GO:0019885 “antigen processing and presentation of endogenous peptide antigen via MHC class I” and GO:0019882 “antigen processing and presentation”). 380 of 451 (84%) cancer genes and 14 of 25 (56%) immune genes were identified with the Ensembl pipeline. An additional 18 cancer genes and 1 immune gene were manually annotated by searching for syntenic regions using the Ensembl “Multi-species view” tool and Exonerate (Slater and Birney, 2005). In order to determine our power for calling variants in the 413 cancer and immune genes, we measured average sequencing depth in the two DFTD genomes and two normal devil genomes across each exon for each gene. We identified 7 exons with average sequence coverage <10 in both of the DFTD genomes, listed below. It is possible that we missed variants in these exons due to lack of sequencing coverage.

*ERCC2*: transcript ID, ENSSHAT00000002476; exon 13
*WAS*: transcript ID, ENSSHAT00000003305; exon 12
*MEN1*: transcript ID, ENSSHAT00000007485; exon 5
*EP300*: transcript ID, ENSSHAT00000007760; exon 23
*AP3D1*: transcript ID, ENSSHAT00000011155; exon 2
*CHEK2*: transcript ID, ENSSHAT00000011864; exon 3
*CDH1*: transcript ID, ENSSHAT00000013098; exon 3
*MYD88*: transcript ID, ENSSHAT00000014458; exon 2

It is also possible that some genes were incompletely annotated due to assembly errors or gaps in the assembly.

**Chromosome Painting**

Tasmanian devil or DFTD chromosomes were analyzed and flow sorted on a flow cytometer (Mo-Flo®, Beckman Coulter) as described previously (Ng et al., 2007; Rabbitts et al., 1995). In brief, 5,000 copies of selected chromosomes were flow sorted separately into sterile 500 μl Eppendorf tubes.

The flow-sorted chromosomes were amplified first using GenomePlex^®^ complete whole genome amplification (WGA) kit (WGA2, Sigma-Aldrich) following the protocol provided by the manufacturer. To make chromosome-specific paint probes, biotin-16-(Roche), Cy5-, Cy3-(Enzo), Green- (Abbotts) and Texas red-12- (Invitrogen) dUTPs were incorporated into the WGA2 products via a round of reamplification using a modified protocol adapted specially for labeling probes. Briefly, for 25 μl of probe labeling reaction, the labeling mix was created by adding 18.2 μl of dH2O, 2.5 μl of 10 × amplification master mix (A5606, Sigma-Aldrich) that came with the WGA3 kit, 2.5 μl of home-made 10 × dNTP mixture containing 2mM each of dATP, dCTP and dGTP, 2mM (dTTP and dUTP), 0.5 μl of 50 mM MgSO_4_, 0.3 μl of BioTaq polymerase (5 U/μl, Bioline), and 1 μl of WGA2 product. The concentrations of dTTP and labeled-dUTP were adjusted according to the labeled-dUTP. For biotin-16-dUTP and Cyanine 3- and Cyanine 5-dUTP, the concentrations of dTTP and dUTP were 1.4 mM dTTP, 0.6 mM dUTP, respectively; for Green-dUTP (Abbotts Molecular), the concentrations of dTTP and dUTP were adjusted to 1.6mM dTTP, 0.4 mM dUTP; for Texas red-dUTP (Abbotts Molecular), 1.8 mM dTTP and 20 mM dUTP. The thermo cycling followed the program suggested by the manufacturer except that the number of thermo cycles was increased from 14 cycles to 18 cycles.

Karyotype analysis was performed according to standard protocols. Metaphase chromosomes were prepared from one fibroblast cell line and four tumor cell lines following the procedure described previously (Yang et al., 1995). The karyotypes were analyzed by a combination of DAPI (4',6-diamidino-2-phenylindole) banding and mutlicolor fluorescence in situ hybridization (FISH) with painting probes as described previously (Yang et al., 1995; Yang and Graphodatsky, 2009). Biotin-labeled probes were detected using Cy5.5- conjugated anti-biotin made in goat (Rockland). Images were captured using a monochrome digital camera (ORCA-EA, Hamamatsu) mounted on an epi-fluorescence microscope (Imager D1, Carl-Zeiss) equipped with narrow bandpass filters specific for Cy5.5, Cy5, Cy3.5, FITC (fluorescein isothiocyanate) and DAPI fluorescence as well as a 200W metal halide light source (Lumen 200, Prior Scientific). The FISH images were processed using SmartCapture® and SmartType softwares (Digital Scientific UK).

## Figures and Tables

**Table 1:** Tasmanian Devil Genome Assembly Features

	Contigs	Supercontigs
Total number	237,291	35,974
Total number of bases	2.93 Gb	3.17 Gb
N50 contig/supercontig size	20,139 bp	1,847,186 bp
Largest contig/supercontig	189,866 bp	5,315,556 bp
Average size	12,354 bp	88,254 bp

See also Table S1 and Figure S1 for further details of Tasmanian devil genome size estimates and Table S2 for a summary of in silico chromosome assignment.

## Accession Numbers

The Tasmanian devil mitochondrial genome has been deposited at DDBJ/EMBL/GenBank under accession JN216828. This Whole Genome Shotgun project has been deposited at DDBJ/EMBL/GenBank under the accession AEFK00000000. The version described in this paper is the first version, AEFK01000000. The genome can be accessed on Ensembl at http://www.ensembl.org/Sarcophilus_harrisii/.

## Supplemental Information

Document S1. Tables S1–S3 and S5

Table S4. Variation Involving Genes in DFTD, Related to Figure 4

Document S2. Article plus Supplemental Information
