# Supplementary material for: Genome Sequencing and Analysis of the Tasmanian Devil and Its Transmissible Cancer
Source: Cell. 2012 Feb 17;148(4):780–91. doi: 10.1016/j.cell.2011.11.065 (PMC3281993; doi:10.1016/j.cell.2011.11.065)
Supplement: Document S1. Tables S1–S3 and S5 [file mmc1.pdf]

Cell, *Volume 148*

Supplemental Information

Genome Sequencing and Analysis of the Tasmanian Devil and Its Transmissible Cancer

Elizabeth P. Murchison, Ole B. Schulz-Trieglaff, Zemin Ning, Ludmil B. Alexandrov, Markus J. Bauer, Beiyuan Fu, Matthew Hims, Zhihao Ding, Sergii Ivakhno, Caitlin Stewart, Bee Ling Ng, Wendy Wong, Bronwen Aken, Simon White, Amber Alsop, Jennifer Becq, Graham R. Bignell, R. Keira Cheetham, William Cheng, Thomas R. Connor, Anthony J. Cox, Zhi-Ping Feng, Yong Gu, Russell J. Grocock, Simon R. Harris, Irina Khrebtukova, Zoya Kingsbury, Mark Kowarsky, Alexandre Kreiss, Shujun Luo, John Marshall, David J. McBride, Lisa Murray, Anne-Maree Pearse, Keiran Raine, Isabelle Rasolonjatovo, Richard Shaw, Philip Tedder, Carolyn Tregidgo, Albert J. Vilella, David C. Wedge, Gregory M. Woods, Niall Gormley, Sean Humphray, Gary Schroth, Geoffrey Smith, Kevin Hall, Stephen M.J. Searle, Nigel P. Carter, Anthony T. Papenfuss, P. Andrew Futreal, Peter J. Campbell, Fengtang Yang, David R. Bentley, Dirk J. Evers, and Michael R. Stratton

Table S1. Tasmanian Devil Genome Size Estimation, Related to Table 1

Method	Estimated Tasmanian devil genome size (Gb)
Number of assembled bases (contigs)	2.93
Number of assembled bases (supercontigs)	3.17
Kmer frequency analysis	3.03
Flow karyotype analysis	2.89
Relative DNA content analysis	2.92

Table S2. ***In Silico*** **Chromosome Assignment , Related to Table 1**

**Chromosome**	**Chromosome DNA content (cytogenetics) (Mb)**	**Number of assigned supercontigs**	**Assigned bases (Mb)**
1	571	6729	684
2	610	8381	740
3	556	7197	641
4	450	4817	487
5	341	3188	300
6	277	2844	263
X	122	2378	86.6
Unassigned		(440)	(1.23)
Total	2927	35,974 (total supercontigs)<br>35,534 (total assigned supercontigs)	3202.8 (Total bases)<br>3201.6 (Total assigned bases)

Table S3. Coordinates of Tumour-Specific Rearrangements, Related to Figure 4
The rearrangements listed were identified and confirmed only in 87T or 53T but not in both tumours.

Sample	Breakpoint 1		Breakpoint 2	
87T	**Coordinate range**	**Strand**	**Coordinate range**	**Strand**
	Chr4_supercontig_000000294: 363864-364141	-	Chr4_supercontig_000000294: 285688-285971	+
	Chr3_supercontig_000000393: 1780799-1781083	-	Chr3_supercontig_000000281: 21827-22022	-
	Chr3_supercontig_000001556: 850-1120	-	Chr3_supercontig_000000315: 147673-147969	+
	Chr5_supercontig_000003149: 550-825	+	Chr2_supercontig_000001430: 157997-158230	+
	Chr4_supercontig_000000654:24128-24412	+	Chr4_supercontig_000000229: 2437030-2437307	+
	Chr2_supercontig_000000207:1235093-1235312	-	Chr2_supercontig_000000207: 1230368-1230615	+
	Chr1_supercontig_000000629: 186664-186891	-	Chr1_supercontig_000000629: 185915-186201	+
	Chr2_supercontig_000000207: 1218849-1219055	-	Chr2_supercontig_000000207: 1214688-1214894	+
	Chr2_supercontig_000000207: 1336201-1336430	+	Chr2_supercontig_000000207: 1335745-1336030	+
	Chr2_supercontig_000000344: 674461-674728	-	Chr2_supercontig_000000344: 672331-672615	-
	Chr1_supercontig_000000182: 1304512-1304801	+	Chr1_supercontig_000000182: 1303661-1303939	+
53T	**Coordinate range**	**Strand**	**Coordinate range**	**Strand**
	Chr3_supercontig_000000191: 233670-233926	-	Chr1_supercontig_000000303: 3376378-3376658	-
	Chr2_supercontig_000000364: 1366493-1366808	+	Chr2_supercontig_000000017: 367364-367641	-
	Chr5_supercontig_000001108: 870-1177	-	Chr1_supercontig_000001069: 40054-40331	-
	Chr1_supercontig_000000253: 91816-92089	-	Chr1_supercontig_000000253: 89326-89611	+
	Chrx_supercontig_000000022: 31467-31764	+	Chrx_supercontig_000000022: 21372-21685	-
	Chr2_supercontig_000000274: 784350-784641	-	Chr2_supercontig_000000274: 739815-740095	+
	Chr3_supercontig_000000130: 1756277-1756562	-	Chr3_supercontig_000000130: 1749479-1749756	+
	Chr1_supercontig_000000356: 1345071-1345388	-	Chr1_supercontig_000000101: 1147092-1147437	-

	Chr5_supercontig_000000097:<br>125064-125382	+	Chr3_supercontig_000000190:<br>1609202-1609504	-
	Chr2_supercontig_000001667:<br>152094-152382	+	Chr2_supercontig_000000584:<br>111379-111671	+
	Chr3_supercontig_000000115:<br>261665-261938	-	Chr3_supercontig_000000115:<br>260047-260297	+
	Chr1_supercontig_000000104:<br>1148801-1149123	-	Chr1_supercontig_000000104:<br>1145329-1145615	+
	Chr5_supercontig_000000152:<br>1991912-1992185	-	Chr2_supercontig_000000061:<br>2384483-2384759	-
	Chr5_supercontig_000000350:<br>74757-75032	-	Chr5_supercontig_000000350:<br>38473-38804	+
	Chr3_supercontig_000000377:<br>2627634-2627909	-	Chr3_supercontig_000000377:<br>2622358-2622613	+
	Chr3_supercontig_000000401:<br>151726-151992	+	Chr3_supercontig_000000393:<br>3853491-3853755	-
	Chr2_supercontig_000000345:<br>100261-100543	+	Chr2_supercontig_000000014:<br>1392972-1393238	+

Table S4. Variation Involving Genes in DFTD, Related to Figure 4
Table is available as an attached Excel spreadsheet.

(A) Non-synonymous single base substitutions in gene exons in 87T and 53T. These variants were found in 87T and 53T but not in either of the normal Tasmanian devil genomes.
(B) Non-synonymous single base substitutions in gene exons in 87T. Variants were found only in 87T but not in 53T or either of the normal Tasmanian devil genomes.
(C) Non-synonymous single base substitutions in gene exons in 53T. Variants were found only in 53T but not in 87T or either of the normal Tasmanian devil genomes.
(D) Indels in gene exons in 87T and 53T. Variants were found in both 87T and 53T or either 87T or 53T but not in either of the normal Tasmanian devil genomes.
(E) Structural variants involving genes in DFTD. Coordinates and gene IDs are listed for structural variants involving genes that were identified in both 87T and 53T but not in either of the normal Tasmanian devil genomes. As confirmation has not been performed for the whole set, this list may include some false positives.

Table S5. Genotyping Summary, Related to Figure 5

The genotypes that define the coloured symbols on the phylogenetic trees in Figure 5 and Figure S4 are shown. The mitochondrial coordinates are based on DDBJ/EMBL/GenBank accession JN216828.

Symbol	Genotype	Type
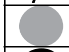	Probable ancestral genotype.	
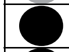	Chr1_supercontig_000000134:1378535C>T	Heterozygous
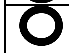	Chr3_supercontig_000000336:2001045T>-<br>Chr1_supercontig_000000248:697762G>A<br>Chr1_supercontig_000000134:1378535C>T	Heterozygous loss<br>Heterozygous<br>Heterozygous
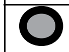	Chr3_supercontig_000000336:2001045T>-<br>Chr2_supercontig_000000158:601261C>T<br>Chr1_supercontig_000000095:2618076T>G<br>Chr1_supercontig_000000248:697762G>A<br>Chr1_supercontig_000000134:1378535C>T<br>MT:1828A>G<br>MT:3255G>A	Heterozygous loss<br>Heterozygous<br>Homozygous<br>Homozygous<br>Heterozygous<br>Mitochondrial<br>Mitochondrial
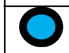	Chr3_supercontig_000000336:2001045T>-<br>Chr2_supercontig_000000158:601261C>T<br>Chr1_supercontig_000000095:2618076T>G<br>Chr1_supercontig_000000248:697762G>A<br>Chr1_supercontig_000000134:1378535C>T<br>MT:1828A>G<br>MT:1928T>C<br>MT:3255G>A	Heterozygous loss<br>Heterozygous<br>Homozygous<br>Homozygous<br>Heterozygous<br>Mitochondrial<br>Mitochondrial<br>Mitochondrial
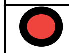	Chr3_supercontig_000000336:2001045T>-<br>Chr2_supercontig_000000158:601261C>T<br>Chr1_supercontig_000000095:2618076T>G<br>Chr1_supercontig_000000248:697762G>A<br>Chr1_supercontig_000000248:403816C>T<br>Chr1_supercontig_000000134:1378535C>T<br>MT:1828A>G<br>MT:3255G>A	Heterozygous loss<br>Heterozygous<br>Homozygous<br>Homozygous<br>Homozygous<br>Homozygous<br>Mitochondrial<br>Mitochondrial
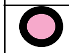	Chr3_supercontig_000000336:2001045T>-<br>Chr2_supercontig_000000158:601261C>T<br>Chr1_supercontig_000000095:2618076T>G<br>Chr1_supercontig_000000248:697762G>A<br>Chr4_supercontig_000000200:4423976T>C<br>Chr3_supercontig_000000295:352495G>A<br>Chr3_supercontig_000000362:528865A>G<br>Chr1_supercontig_000000248:403816C>T<br>Chr1_supercontig_000000134:1378535C>T<br>MT:1828A>G<br>MT:3255G>A<br>MT:2572G>A	Heterozygous loss<br>Heterozygous<br>Homozygous<br>Homozygous<br>Heterozygous<br>Homozygous<br>Homozygous<br>Heterozygous<br>Homozygous<br>Mitochondrial<br>Mitochondrial<br>Mitochondrial
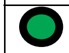	Chr3_supercontig_000000336:2001045T>-<br>Chr2_supercontig_000000158:601261C>T<br>Chr1_supercontig_000000095:2618076T>G<br>Chr1_supercontig_000000248:697762G>A<br>Chr1_supercontig_000000134:1378535C>T<br>MT:1828A>G<br>MT:3430T>A<br>MT:4311G>A<br>MT:3255G>A	Heterozygous loss<br>Heterozygous<br>Homozygous<br>Homozygous<br>Heterozygous<br>Mitochondrial<br>Mitochondrial<br>Mitochondrial<br>Mitochondrial
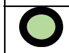	Chr3_supercontig_000000336:2001045T>-<br>Chr2_supercontig_000000158:601261C>T<br>Chr1_supercontig_000000095:2618076T>G<br>Chr1_supercontig_000000248:697762G>A<br>Chr1_supercontig_000000134:1378535C>T<br>MT:1828A>G<br>MT:2731A>G<br>MT:3430T>A	Heterozygous loss<br>Heterozygous<br>Homozygous<br>Homozygous<br>Heterozygous<br>Mitochondrial<br>Mitochondrial<br>Mitochondrial

	MT:4311G>A<br>MT:3255G>A	Mitochondrial<br>Mitochondrial
	MT:7198T>C	Mitochondrial
	MT:12288C>T<br>MT:7198T>C	Mitochondrial<br>Mitochondrial
	MT:3161G>A	Mitochondrial
	MT:3161G>A<br>MT:14209A>T	Mitochondrial
	MT:2707G>A	Mitochondrial
	MT:2707G>A<br>MT:3679G>A	Mitochondrial
	MT:6745T>C	Mitochondrial
	MT:14114C>T	Mitochondrial
	MT:2942C>T	Mitochondrial
	MT:4266G>A	Mitochondrial
	MT:2261T>C	Mitochondrial
	Chr1_supercontig_000000095:591062C>A<br>Chr2_supercontig_000000205:1697548C>T<br>Chr1_supercontig_000000246:95466A>G<br>Chr2_supercontig_000000205:4043679A>T	Homozygous<br>Homozygous<br>Homozygous<br>Homozygous
	Chr1_supercontig_000000095:591062C>A<br>Chr2_supercontig_000000205:1697548C>T<br>Chr1_supercontig_000000246:95466A>G<br>Chr2_supercontig_000000205:4043679A>T<br>MT:10133C>T	Homozygous<br>Homozygous<br>Homozygous<br>Homozygous<br>Mitochondrial
	Chr3_supercontig_000000336:2001045T>-<br>Chr2_supercontig_000000158:601261C>T<br>Chr1_supercontig_000000095:2618076T>G<br>Chr1_supercontig_000000248:697762G>A<br>Chr4_supercontig_000000200:4423976T>C<br>Chr3_supercontig_000000295:352495G>A<br>Chr3_supercontig_000000362:528865A>G<br>Chr1_supercontig_000000248:403816C>T<br>Chr1_supercontig_000000134:1378535C>T<br>MT:1828A>G<br>MT:3255G>A<br>MT:2572G>A<br>MT:611C>T	Heterozygous loss<br>Heterozygous<br>Homozygous<br>Homozygous<br>Heterozygous<br>Homozygous<br>Homozygous<br>Heterozygous<br>Homozygous<br>Mitochondrial<br>Mitochondrial<br>Mitochondrial<br>Mitochondrial
	Chr3_supercontig_000000336:2001045T>-<br>Chr2_supercontig_000000158:601261C>T<br>Chr1_supercontig_000000095:2618076T>G<br>Chr1_supercontig_000000248:697762G>A<br>Chr1_supercontig_000000134:1378535C>T<br>MT:1828A>G<br>MT:3255G>A<br>MT:4422G>A	Heterozygous loss<br>Heterozygous<br>Homozygous<br>Homozygous<br>Heterozygous<br>Mitochondrial<br>Mitochondrial<br>Mitochondrial
	Chr1_supercontig_000000095:591062C>A<br>Chr2_supercontig_000000205:1697548C>T<br>Chr1_supercontig_000000246:95466A>G<br>Chr2_supercontig_000000205:4043679A>T<br>MT:10133C>T<br>Chr2_supercontig_000000031:817867C>T	Homozygous<br>Homozygous<br>Homozygous<br>Homozygous<br>Mitochondrial<br>Heterozygous
